# Strategically Patrolling in a Chemical Cluster Addressing Gas Pollutants’ Releases through a Game-Theoretic Model

**DOI:** 10.3390/ijerph16040612

**Published:** 2019-02-20

**Authors:** Bin Chen, Zhengqiu Zhu, Feiran Chen, Yong Zhao, Xiaogang Qiu

**Affiliations:** College of System Engineering, National University of Defense Technology, Changsha 410073, China; nudtcb9372@gmail.com (B.C.); 15507497024@163.com (F.C.); zhaoyong15@nudt.edu.cn (Y.Z.); Michael.qiu@139.com (X.Q.)

**Keywords:** chemical cluster environmental protection patrolling game, source estimation process, patrolling strategy, fixed route strategy, purely randomized route strategy

## Abstract

Chemical production activities in chemical clusters, if not well managed, will pose great threats to the surrounding air environment and impose great burden on emergency handling. Therefore, it is urgent and substantial in a chemical cluster to develop proper and suitable pollution controlling strategies for an inspection agency to monitor chemical production processes. Apart from the static monitoring resources (e.g., monitoring stations and gas sensor modules), patrolling by mobile vehicle resources is arranged for better detecting the illegal releasing behaviors of emission spots in different chemical plants. However, it has been proven that the commonly used patrolling strategies (i.e., the fixed route strategy and the purely randomized route strategy) are non-optimal and fail to interact with intelligent chemical plants. Therefore, we proposed the Chemical Cluster Environmental Protection Patrolling (CCEPP) game to tackle the problem in this paper. Through combining the source estimation process, the game is modeled to detect the illegal releasing behaviors of chemical plants by randomly and strategically arranging the patrolling routes and intensities in different chemical sites. In this game-theoretic model, players (patroller and chemical sites), strategies, payoffs, and game solvers are modeled in sequence. More importantly, this game model also considers traffic delays or bounded cognition of patrollers on patrolling plans. Therefore, a discrete Markov decision process was used to model this stochastic process. Further, the model is illustrated by a case study. Results imply that the patrolling strategy suggested by the CCEPP game outperforms both the fixed route strategy and the purely randomized route strategy.

## 1. Introduction

The so-called chemical clusters are formed due to economies of scale, environmental factors, and other collaboration benefits (e.g., social motives and legal requirements) [1]. However, within such clusters, the situation is that the air pollutants produced in the process of chemical production are often illegally released to the surrounding environments instead of being purified and treated [2]. In extreme situations, the accidental releases caused by spontaneous or anthropogenic activities can exert dramatic implications [3]. Through transportation and dispersion (e.g., atmospheric flow), humans and the environment can be undesirably exposed to these dangerous gas emissions and eventually suffer from the harmful or fatal effects. Therefore, in order to avoid the release of gas substances of high toxicity and ensure the protection of environment and human health, air quality monitoring and gas emission detection have to be performed regularly [4]. At present, a core issue of concern to those who manage the chemical cluster is the effective prevention and mitigation of impacts caused by risk accidents and the implementation of effective management that can ensure safe production and social stability [5]. 

To this end, Qiu et al. [6] proposed a method incorporating a drone-based monitoring platform and a source estimation method to estimate contaminant sources in a chemical cluster. This research work falls through providing practical and efficient patrolling routes for mobile inspection resources hereafter. Fortunately, game theory has the advantage on modeling limited resource deployment in a multiple stakeholders’ situation through a sound mathematical approach, according to Tambe et al. [7]. Namely, one of the applications of game theory in the security domain is to schedule patrolling (i.e., an act of traveling of mobile resources at different locations and intervals) [8] and this kind of patrol is also introduced in some other domains.

The security games have been successfully deployed in many areas (airports [9], ports [10], and trains [11]) to protect infrastructures for randomizing schedules for patrolling and monitoring. Also, a game-theoretic concept is deployed by Aguirre et al. [12] to define a multi-agent patrolling strategy on a national border to achieve a safer country. Similar works can be found in these references, such as Basilico et al. [13] and Gatti [14]. The zero-sum graph patrolling games defined by Alpern et al. [15,16] and Papadaki et al. [17] can perfectly solve the game on some special graphs, such as the line graph, etc. However, the defined zero-sum graph patrolling games are unreasonable in many situations and in their line graph patrolling game, the quantitative risk assessment of the line was absent [18]. Subsequently, to solve this problem, the Pipeline Patrolling Game (PPG) was proposed by Amirali et al. [19] in the Bayesian Stackelberg game form based on security risk assessment. Other than in security domain, scheduling patrol also sprung up in environmental protection domain in recent years. Green Security Games (GSGs) are typical representatives, applications of which mainly focused on scheduling patrols to protect the forests, fish, wildlife, etc. [20]. However, few works have realized the importance of scheduling patrols in a chemical cluster to detect the spontaneous or anthropogenic industrial production emission activities intelligently.

Different from the above mentioned game-theoretic applications in the security domain [7,21] and in the environment domain [22,23,24,25], Zhu et al. [26,27] proposed the Chemical Plant Environmental Protection (CPEP) game and the extended CPEP game in succession, which were the first works for optimizing audits and detections of illegal release of atmospheric contaminants in chemical clusters. In these models, the game-theoretic model in conjunction with source estimation methods was utilized to better schedule the static inspection resources (i.e., high-accuracy monitoring stations and gas sensor modules) for detecting the irregularities of chemical plants. Besides the static monitoring resources, mobile inspection resources are highly recommended to monitor the chemical production process for their flexibility and mobility. In this paper, the Stackelberg game is therefore applied for scheduling the patrol of mobile inspection resources in a chemical cluster. For analyzing the patrolling in a chemical cluster, the patrolling object is modeled as a graph, in which the nodes are different chemical sites in the cluster. The game-theoretic model involves intelligent interactions between patrol teams (i.e., the defender) and chemical plants (i.e., many attackers). To model this interaction precisely and practically, several challenges and uncertainties remain to be solved. (i) It is unavoidable to face the challenge of large state space to represent strategies for the players since the game takes place on a road network. (ii) The patroller would travel in the graph and stay inside some nodes for a certain period of time and implement inspections to detect illegal releases of chemical plants through source estimation methods. However, the strategy generated by our model cannot guarantee the 100% success rate of catching the violations of chemical plants. (iii) Due to traffic delays or some other cognitive reasons, the patroller may not follow the patrolling schedules precisely. Thus, the present paper proposes a Chemical Cluster Environmental Protection Patrolling (CCEPP) game, answering the question of how to optimally randomize patrolling in a chemical cluster and deal with the aforementioned challenges and uncertainties. In this way, the proposed method not only facilitates the decision-making process of a patrolling route for the patroller team, but also addresses the atmospheric pollutants controlling problem, as well as reduces the risk of accidental gaseous pollutant leaks. 

The remainder of the paper is organized as follows: Section 2 deals with the road network modeling problem, the CCEPP game and its corresponding game solver are proposed in Section 3, Section 4 involves an illustrative case study and experimental results, and conclusions and future directions are drawn in Section 5.

## 2. Road Network Modeling

A typical patrolling scenario can be demonstrated as follows: Patrol teams drive the inspection vehicles randomly, patrolling inside each of the chemical sites or travelling on the public road to another chemical site. During patrolling inside a chemical site, the team would utilize the monitoring facilities to collect atmospheric data for source estimation. Besides monitoring data from static inspection resources (i.e., monitoring stations and gas sensor modules), if the patrol team is patrolling inside this chemical site when some of the emission sources in this site are releasing excessive atmospheric contaminants, then the emission sources certainly have a probability of being detected by the patrol team. After staying for a period of time, the patrol team would travel to another site via the connected road. Therefore, to depict such a patrolling process, which is changing with space-time, road network is definitely modeled first.

### 2.1. Graphic Modeling

Several criteria are provided to determine the nodes and edges in a graph based on the practical road network: (i) If a chemical site only has one chemical plant, it usually owns one vehicle entrance; (ii) if a chemical site has several chemical plants and each chemical plant has several emission sources, we can assume that each chemical plant in this site has a vehicle entrance, all of which are assumed to be fully connected as well; (iii) the vehicle entrances are usually located on the side of public road; (iv) if two entrances belonging to different sites cannot be connected through a straight segment, a crossroad has to be added. Therefore, we model the road network as a graph G(*V,E*), where *V* denotes the number of nodes of the graph (i.e., the vehicle entrances of each site and the crossroads), and *E* is the number of edges of the graph (i.e., the roads between different nodes). 

**Figure 1:**
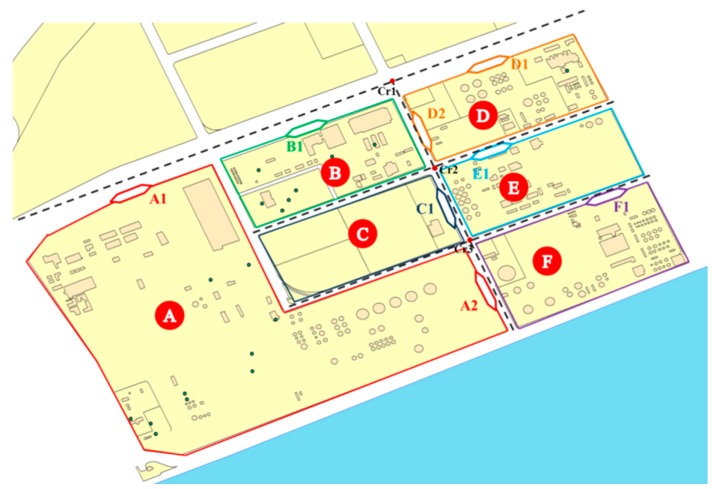
Layout of part of a chemical cluster in Shanghai.

**Figure 2:**
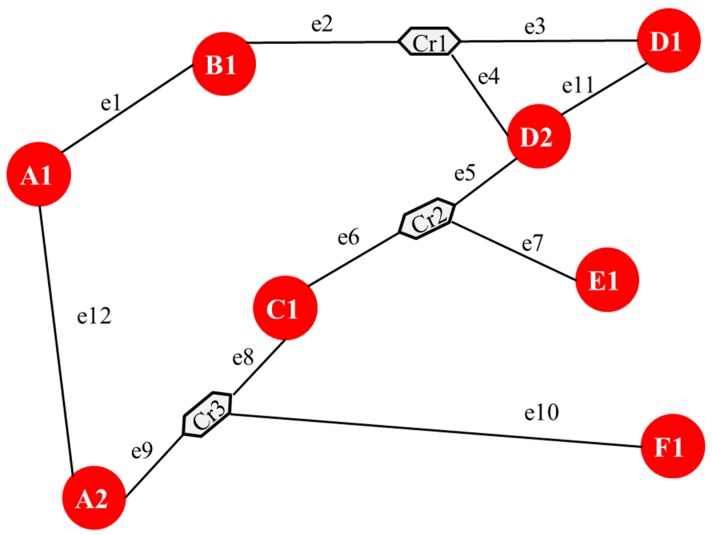
Graphic modeling of this chemical cluster.

For the sake of illustration, an example of a small part of the Shanghai chemical cluster is given. There are six chemical sites in this picture, indexed as site ‘A’, site ‘B’, and so forth. As we may notice, chemical sites ‘A’ and ‘D’ have two vehicle entrances, while other chemical sites have only one entrance. Moreover, three crossroads are added to link two sites which cannot be connected through a straight segment. We used black dotted lines in this figure to demonstrate the traffic roads in reality for the patroller to drive. Meanwhile, based on above mentioned criteria, the graphic model of this cluster shown in Figure 1 was displayed in Figure 2. As we can notice in this picture, nodes in Figure 2 are represented by the sites’ entrances (i.e., ‘A1’, ‘A2’, ‘B1’, ‘C1’, ‘D1’, ‘D2’, ‘E1’, ‘F1’) and the crossroads (i.e., ‘Cr1’, ‘Cr2’, ‘Cr3’). Further, edges ‘e1’ to ‘e12’ are constructed to reflect the vehicle roads based on actual connection relationships of these nodes.

Based on the graphic model, we illustrate the graphic patrolling problem of mobile vehicles as follows: (i) A patroller (or several patrol teams) starts her patrolling from a node (i.e., the dummy source node); (ii) she moves on the nodes and edges in the graph; (iii) when arriving at a node (a chemical site), she may decide whether to stay at the node for a specific period of time $t_{i}^{p}$ (inspect inside the site), or move to another site instantly with a period of time $t_{e}^{m}$, without patrolling the current site; (iv) after the maximum travelling time budget of a patrol *T* is expended, the patroller terminates the patrolling and goes back to the dummy source node.

In the above statement, $t_{i}^{p}$ denotes the inspection time in a site *i*; $t_{e}^{m}$ represents the travelling time on an edge *e*; and *T* denotes the maximum travelling time budget of a patrol. For the sake of simplicity, the periods of time $t_{i}^{p}$ and $t_{e}^{m}$ are assumed to be fixed values in this paper, though they can be influenced by different entrances from the same site and multiple patrolling intensities. 

With the definitions of inspecting time $t_{i}^{p}$ and travelling time $t_{e}^{m}$, we can define a superior connection matrix *sC* of graph *G*. Based on the superior connection matrix, an algorithm is proposed in Section 2.3 to construct a transition graph. An entry sC(i,j) in matrix *sC* represents the time cost through moving from node *i* to node *j* (of graph *G*). There are two possible scenarios regarding the relationship of nodes *i* and *j*: (i) The two nodes belong to different chemical sites or at least one of them is a crossroad node. In this case, sC(i,j) is equal to the travelling time $t_{e}^{m}$ that the patroller needs to move from node *i* and node *j*; or (ii) the two nodes belong to different entrances of a chemical site, or are the same. In this situation, sC(i,j) is equal to the inspection time $t_{i}^{p}$ of the site.

Then, for the cluster and graph illustrated in Figure 1 and Figure 2, if we set $t_{1}^{m}$ = 4, $t_{2}^{m}$ = 2, $t_{3}^{m}$ = 3, $t_{4}^{m}$ = 1, $t_{5}^{m}$ = 1, $t_{6}^{m}$ = 2, $t_{7}^{m}$ = 2, $t_{8}^{m}$ = 2, $t_{9}^{m}$ = 2, $t_{10}^{m}$ = 3, $t_{11}^{m}$ = 3, $t_{12}^{m}$ = 6, and further set $t_{A}^{p}$ = 10, $t_{B}^{p}$ = 6, $t_{C}^{p}$ = 5, $t_{D}^{p}$ = 7, $t_{E}^{p}$ = 6, $t_{F}^{p}$ = 7 (the exact values are determined by road distance and driving speed), Table 1 shows an example of this matrix by using the values mentioned above. For instance, $t_{1}^{m}$ is the driving time from node ‘A1’ to ‘B1’ and $t_{A}^{p}$ denotes the time needed to inspect inside the chemical site ‘A’. All the time-related data are unified in minutes. 

### 2.2. Time Discretization

To simplify the transition graph model, a discretization of the time is necessary. We discretize time into an equal granularity of *h* minutes (i.e., the time slice is determined by practical conditions and is denoted as one minute in this study, however, it can also be one second or one hour as well). In this way, a time vertex is added every *h*-minute until the maximum inspection time budget *T* has been expended. Moreover, if the time dimension of the transition graph is continuous, the patroller’s travelling time, the patroller’s patrolling time, and the attacker’s attack period are not necessarily integers. Thus, these time-related parameters should be rounded to their closest integer numbers of time slice in order to discretize the time dimension. Consequently, any actions of the patroller and the attacker would happen at the beginning of the vertex of each time slice.

Although the time dimension of the transition graph is continuous in reality, such a discretization mentioned above is also reasonable and feasible. For one thing, the attacker’s actions can be enumerated by discretizing the time, as seen in Section 3.1. For another thing, if the length of a time slice is short enough, the discretization model can also describe the reality well.

### 2.3. Transition Graph Modeling

A transition graph tG(tV,tE) is defined based on the graphic model of a chemical cluster and the time discretization. A node (or state) in tG is denoted by a tuple of (t,i), wherein t∈[0,T) represents the current time step and i∈{1,2,…,|V|} denotes the site that the patroller is located (it is also denoted as a node in graph G(V,E)). After choosing an action and moving to another site $i_{2}$ at time $t_{2}$ from current site $i_{1}$ at time $t_{1}$, a directed edge in tG is connected between the two node $\left(t_{1},i_{1}\right)$ and $\left(t_{2},i_{2}\right)$.

In the transition graph, we need only enumerate a polynomial number of nodes (or states) and edges instead of enumerating an exponential number of pure strategies. The goal of this paper is to compute the optimal probability flow (i.e., marginal coverage vector) and sample from this vector to create inspection schedules for the inspection agency. However, due to traffic delays or some other cognitive reasons, the patroller may not follow the patrolling schedules precisely. We therefore have to incorporate this uncertainty into the transition graph. Fortunately, Markov decision processes (MDPs) provide a mathematical paradigm for modeling decision making in situations where outcomes are partly random and partly under the control of a decision maker. In our situation, a discrete MDP can model the discrete time stochastic control process of the actions and states. To be specific, the process is in a state *s* at each time step, and the decision maker may choose any available action *a* in state *s*. At the next time step, the process would move into a new state *e* randomly at a certain probability $P_{a}(s,e)$, giving a reward $R_{a}(s,e)$ correspondingly. Therefore, a discrete MDP in this paper is represented by a 3-tuple $\left(S,A,P_{a}(s,e)\right)$ without considering the discount factor *γ* and the immediate reward $R_{a}(s,e)$.

*S* is a finite set of states. Each state s∈S is a tuple (t,i). Each vertex tv∈tV in the transition graph corresponds to a state *s* and thus *S* is equal to *tV*;

*A* is a finite set of actions which corresponds to the set of actions available from current state *s*, i.e., the set of sites connected by site *i*;

$P_{a}(s,e)=pro(s_{t+1}=e|s_{t}=s,a_{t}=a)$ represents the probability that the patroller takes action a in state *s*, leading to state e.

**Figure 3:**
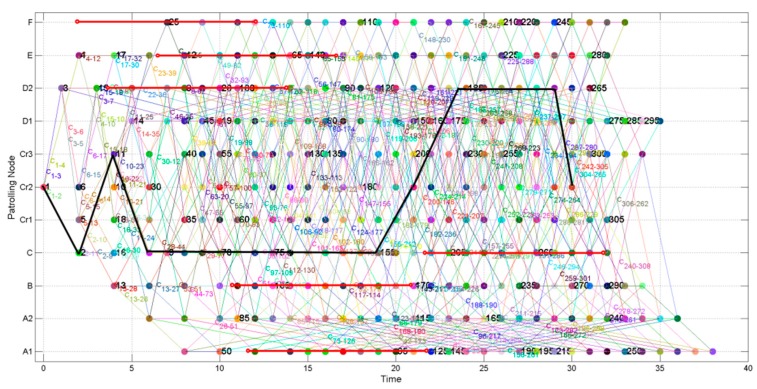
The illustrative example of the transition graph.

Table 2 shows an iterative algorithm for generating the transition graph tG(tV,tE). The essence of this algorithm is to find all the connective nodes of each node in the graphic model. In this table, dis(dsn,nd) denotes the shortest distance in graph *G* from the dummy source node dsn to node nd. An example of the patrolling graph *tG* for the chemical cluster with the data of patrolling and inspecting time in Table 2 is shown in Figure 3 to further illustrate how the algorithm works. We further assume the inspection time budget *T* = 30 and dummy source node close to the ‘Cr2’.

In Figure 3, the *x* axis and the *y* axis denote the time dimension and the nodes in graphic model, respectively. Therefore, a possible node for *tG* can be any coordinates in this figure. As we may notice, node 1 (at the left-hand side of this figure) is (0, ‘Cr2’), which means that at time 0, the patroller starts from her source node (i.e., ‘Cr2’). Thereafter she has three choices: (i) To come to site ‘C’ with a driving time $t_{6}^{m}$ and reach node 2 (i.e., (2, ‘C’)); (ii) to come to site ‘D’ (more accurately, entrance ‘D2’) with a driving time $t_{5}^{m}$ and reach node 3 (i.e., (1, ‘D2’)); (iii) to come to site ‘E’ with a driving time $t_{7}^{m}$ and reach node 4 (i.e., (2, ‘E’)). Subsequently, at these new nodes (e.g., 2, 3, or 4), the patroller has to face the same choice problem (i.e., to patrol the current chemical site or to come to the adjacent chemical sites). Finally, when the total patrol time satisfies, the patroller terminates the patrol and comes back to her source node. In Figure 3, indexes of some nodes are marked to clarify this patrolling problem. 

In Figure 3, a fixed patrolling route can be demonstrated as a series of edges $\left(te^{1},te^{2},\ldots ,te^{len}\right)$ satisfying the following three conditions in the transition graph: (i) The in-degree of the start node of $te^{1}$ is 0; (ii) the out-degree of the end node of $te^{len}$ is 0; (iii) the edges $te^{j}$ and $te^{j+1}$ (j=1,2,…,len−1) are linked, which means that the end node of $te^{j}$ is the start node of $te^{j+1}$. For instance, the bold and black line in Figure 3 refers to a fixed patrolling route, and it is: ‘Cr2’ → ‘C’ → ‘Cr3’ → ‘C’ → patrol chemical site ‘C’ → ‘Cr2’ → ‘D2’ → patrol chemical site ‘D’ → back to ‘Cr2’. A purely randomized patrolling route can be defined as: “At any node of the transition graph, the patrol team goes to each edge outgoing from the node with an equal probability.” For example, in this figure, when the patroller is at node 1 (0, ‘Cr2’), she would go to node 2, 3 or 4 with an equal probability of 1/3, and so forth. 

In this paper, the patroller is required to prolong her patrolling in the chemical site to keep the continuity of coverage on each chemical site until the next patroller might be able to arrive at the site (see step 4.2.2 in Table 2). For instance, in Figure 3, the maximum patrolling time budget is set as 30 min, however, the patrolling in chemical site ‘A’ is not stopped until 38 min. The idea is that the shortest time in which the next patrolling team can arrive at chemical site ‘A’ from source node ‘Cr2’ is 8 min. However, if the current patroller does not prolong her patrolling in this chemical site and the next patrol team starts at time 30 from the source node ‘Cr2’, then chemical site ‘A’ would definitely not be inspected during that time interval between 30 and 38. This method may increase the patroller’s workload. However, the problem can be solved if we set the value of *T* slightly smaller than the patroller’s real workload.

## 3. Chemical Cluster Environmental Protection Patrolling Game

The CCEPP game is proposed in this section to model practical interactions between the patroller and the chemical plants. Then, we introduce the game from four aspects in succession, including players modeling, strategy modeling, payoff modeling, and the solutions of the game.

### 3.1. Players Modeling

Players of the CCEPP game are the patrol teams (i.e., mobile inspection resources, such as vehicles, helicopters, or drones) on the one hand and the chemical plants on the other. Hereafter, patrol teams are referred to as “leader” or “defender” and the chemical plants are referred to as “follower” or “attacker”. The aim of the defender is to optimize the patrolling plans of mobile vehicles to detect more irregularities of chemical plants and to improve her payoff in the meantime. After observing the actions taken by the defender, the attacker attempts to release excessive air pollutants to optimize his profits. Moreover, both players in this game are assumed with perfect rationality based on two basic reasons: One is that both players in the CCEPP game are able to perceive their situation and the opposite player’s actions accurately, and the other is that the players tend to maximize their payoffs through intelligently planning their strategies. Future work can be devoted to extend the model to deal with boundedly rational attackers.

### 3.2. Strategy Modeling

#### 3.2.1. Attacker’s Strategy

To simplify the modeling of the attacker’s strategy, several reasonable assumptions are made: (i) The number of working release spots differs from those in different release scenarios; (ii) in a chemical site, *i*, the release spots have the same working start time and the same working duration; (iii) the working duration of the release spots from different chemical sites are usually different because the categories of atmospheric contaminants are different within different chemical sites. Therefore, three parts are involved in an attacker’s strategy: (i) Determine a time to start the release; (ii) determine a release scenario to use (denoted by the working duration of release spots); and (iii) determine the number of release spots to use. 

Specifically, the formulation of the attacker’s pure strategy is listed in Formula (1).

(1) $$s_{a}^{i}=(t,k_{i},rs_{i}),$$

where t represents the releasing start time, $k_{i}$ denotes the working duration of release spots (e.g., five minutes), and $rs_{i}$ is the number of working release spots (this number is a positive integer between 1 and the total number of release spots in a site). As can be noticed in Figure 3, there is a red and bold line in each chemical site representing one attack scenario of attackers. For instance, the red and bold line in chemical site ‘F’ represents a release of site ‘F’ starting at time 2, with a release duration of 10 min.

Formula (1) implies that each chemical site would choose one attack scenario to implement. Moreover, we can compute the number of pure strategies for a site *i* through Formula (2) based on the above definitions, in which $\left|S_{a}^{i}\right|$ is the number of pure strategies owned by the attacker; $\bar{T}$ denotes the total time slices based on the segment of the maximum inspection budget; Sce is the number of different release scenarios; and $RS_{i}$ represents the total number of release spots owned by a site *i*.

(2) $$|S_{a}^{i}|=\bar{T}\cdot Sce\cdot (2^{RS_{i}}-1),$$

#### 3.2.2. Defender’s Strategy

As illustrated in the definition of transition graph, a flow through the graph corresponds to a specific defender patrolling strategy and that flow is represented by a marginal coverage vector $\Pi_{(s,e)\in tE}c_{a}(s,e)$. To be specific, two different states in the transition graph are connected by an edge. Thus, the marginal probability means that the inspection resources may go that edge. Moreover, once the optimal flow is computed, we can sample from which to generate the random patrol schedules. Here, the defender’s strategy is denoted as Formula (3).

(3) $$s_{d}=\Pi_{(s,e)\in tE}c_{a}(s,e),$$

where $c_{a}(s,e)$ denotes the marginal coverage probability of the patroller assigned to the edge from node s to node e (reach state s, execute action a, and end up in state e); and Π is the Cartesian product of all edges in tG (i.e., all (s,e)∈tE).

Further, the marginal probability of the inspection resources reaching state *s* and executing action a is defined as $\omega_{a}(s)$. We then define a dummy source node $s^{+}$ to represent a root node which has no income edges, while a dummy sink node $s^{-}$ is defined to represent a terminal node end at the maximum inspection time budget *T* with only income edges. An intermediate node of tG is a node that has both income edges and outcome edges. Moreover, two properties should be satisfied based on the graphic flow theory: (i) For each intermediate node, the sum of all the income probabilities must equal the sum of all the outcome probabilities (i.e., the flow into a state s is equal to the flow out of the state); (ii) the sum of probabilities coming out from the root node or coming into the terminal node corresponds to the quantity of mobile inspection resources. Formulas (4) and (5) illustrate the abovementioned two equalities.

(4) $$\sum_{in\in \{e\in tV|(e,s)\in tE\}}c_{a^{\prime }}(in,s)=\sum \omega_{a}(s)=\sum_{out\in \{o\in tV|(s,o)\in tE\}}c_{a}(s,out),$$

(5) $$\sum_{out\in \{o\in tV|(s^{+},o)\in tE\}}c_{a^{\prime }}(s^{+},out)=\sum \omega_{a^{\prime }}(s^{+})=\sum_{in\in \{e\in tV|(e,s^{-})\in tE\}}c_{a}(in,s^{-})=r,$$

Moreover, due to actual traffic delays or a patroller’s bounded cognition on patrolling plans, the same action a taken by a patroller from a state s may not lead to a fixed state *e*. The following equation is used to explain the relationship between the marginal probabilities and state transition probabilities. It defines that marginal coverage probability $c_{a}(s,e)$ equals to the multiplication of the marginal probability $\omega_{a}(s)$ and the probability of successfully transitioning to state e.

(6) $$c_{a}(s,e)=\omega_{a}(s)P_{a}(s,e)\forall s,e\in tV,$$

### 3.3. Payoff Modeling

In this paper, the owner of a chemical site is assumed to choose an attack scenario to perform. Further, there will be two possible results when he chooses to attack, being: (i) The attack fails, or (ii) the attack is successfully implemented. In case the attack succeeds, the patroller will suffer a loss $L_{d}^{i}$ (from the pressure of public opinion and authorities) and the attacker will obtain a gain $G_{a}^{i}$ (from releasing excessive air contaminants without purification treatment). If the attack fails, the patroller will acquire a reward $R_{d}^{i}$ and the attacker will suffer a penalty $P_{a}^{i}$. Both reward $R_{d}^{i}$ and penalty $P_{a}^{i}$ come from forfeit of attackers. Also, an expenditure $r\cdot C_{d}$ is defined to represent the cost for conducting a patrolling through the chemical cluster. Values used in this paper of all these parameters are determined by experts from the environmental protection authorities of Shanghai’s chemical cluster. Then, the defender’s and attacker’s payoff are formulated in Formulas (7) and (8), wherein $f(\tilde{f})$ is the probability that the attack would fail from the defender’s (the attacker’s) perspective and $\rho^{i}$ is the prior probability.

(7) $$u_{d}=\sum_{i=1}\{\rho^{i}\cdot [R_{d}^{i}\cdot f-L_{d}^{i}\cdot (1-f)]\}-r\cdot C_{d},$$

(8) $$u_{a}^{i}=[G_{a}^{i}(1-\tilde{f})-P_{a}^{i}\cdot \tilde{f}],$$

In Formulas (7) and (8), $f(\tilde{f})$ is a variant related to the attacker’s strategy and defender’s strategy modeled in Section 3.2. In the following paragraphs, the calculation of the probability $f(\tilde{f})$ is studied.

The probability that the irregularities of chemical sites would be detected by the static inspection resources (i.e., monitoring stations and gas sensor modules) is denoted by parameter $f_{cpep}$ and can be computed through the Chemical Plant Environmental Protection (CPEP) game or can be assessed by environmental experts as well. Moreover, we represent the probability that the patroller would detect the irregularities of chemical sites successfully as parameter $f_{p}$. Considering the case that an excessive release can be detected by mobile resources and static resources at meantime, we therefore formulate the probability $f(\tilde{f})$ as Formula (9).

(9) $$f=f_{p}+f_{cpep}-f_{p}\cdot f_{cpep}=1-(1-f_{cpep})\cdot (1-f_{p}),$$

In Formula (9), $f_{cpep}$ is a site-specific constant. Taking into the characteristics of source estimation methods [28,29,30,31], we selected two main factors to model the probability $f_{p}$. A release in site *i* starts at time *t* and lasts for $k_{i}$ time slices with $rs_{i}$ release spots. If a selected patrol of a defender has certain marginal coverage probabilities assigned in site *i* after the release time *t*, the inspection resources have a certain amount of time to collect the release date used for source estimation. Moreover, the quantity of working release spots in an attack scenario significantly influences the detection probabilities. Generally speaking, the probability $f_{p}$ would decrease when the quantity of working release spots increases. 

Furthermore, we define an effective data collecting time as $t_{eff}$, which is constituted of two components: $t_{overlap}$ and $t_{after}$. The time $t_{overlap}$ means that the data collecting time is located on the overlaps between the release procedure of chemical sites and the patrollers staying in the site. Meanwhile, the time $t_{after}$ represents that the data collecting time is located after the release finishing time. Based on the characteristic of source estimation method, pollution data collected during the overlap time is more useful than that collected after the release finishing time. Therefore, the effective data collecting time can be formulated as Formula (10).

(10) $$t_{eff}=\varepsilon \cdot t_{overlap}+t_{after},$$

in which ε is a real number larger than 1 and $t_{overlap}$ can be represented by $\left[\max\{t,st\},\min\{t+k_{i},st+t_{i}^{p}\}\right]$. If we denote the start time that the patroller stays in a site *i* as st, the staying behavior of the patroller can be represented by a tuple of starting time and staying duration $\left(st,t_{i}^{p}\right)$. There are five situations classified to calculate the effective data collecting time $t_{eff}$.

**Situation** **1:** *If $st+t_{i}^{p}\leq t$ holds, it means that the release scenario has not started. In this situation, the effective data collecting time $t_{eff}$ equals 0.*

**Situation** **2:** *If $st<tandst+t_{i}^{p}>t\;and\;st+t_{i}^{p}\leq t+k_{i}$ holds, it means that only the overlap time is the data collecting time. In this situation, the effective data collecting time $t_{eff}$ equals $\varepsilon \cdot t_{overlap}$.*

**Situation** **3:** *If $st>t\;and\;st+t_{i}^{p}\leq t+k_{i}$ holds, it also means that only the overlap time is the data collecting time. In this situation, the effective data collecting time $t_{eff}$ equals $\varepsilon \cdot t_{overlap}$.*

**Situation** **4:** *If $st>t\;and\;st+t_{i}^{p}>t+k_{i}$ holds, it means that the total data collecting is patrolling time $t_{i}^{p}$ in this site. In this situation, the effective data collecting time $t_{eff}$ equals $t_{eff}=\varepsilon \cdot t_{overlap}+t_{after}$, in which $t_{after}$ equals $t_{i}^{p}-t_{overlap}$.*

**Situation** **5:** *If $st>t+k_{i}$ holds, it also means that the total data collecting is patrolling time $t_{i}^{p}$ in this site. But in this situation, the effective data collecting time $t_{eff}$ equals $t_{after}$.*

After listing these situations, the probability, $f_{p}$, can be formulated through Formula (11).

(11) $$f_{p}=\sum_{sit=1}\sigma_{sit}\cdot \sum_{s\in S_{i}^{sit}}\omega_{a}(s),$$

in which $\sigma_{sit}$ is a parameter with respect to effective data collecting time $t_{eff}$ and working release spots $rs_{i}$, denoted by Formula (12). In Formula (12), $\sigma_{i}$ is a positive, real number related to detection probability. $S_{i}^{sit}$ represents the states set satisfying the specific situation (the states set relates to all time steps associated with site *i*). Based on Formula (4), $\sum_{s\in S_{i}^{sit}}\omega_{s}(a)$ can be represented by a polynomial summation of marginal coverage vector $\vec{c}$.

(12) $$\sigma_{sit}=\sigma_{i}\cdot (t_{eff}/t_{i}^{d})\cdot (RS_{i}/rs_{i}),$$

### 3.4. Game Solver and Solution Definition

In the CCEPP game, it is assumed that the attacker can collect information about the patroller’s patrolling routes. For instance, the attackers would acquire the information regarding the patrolling route through long-term observation on patrolling teams or stealing the patrolling plans. Therefore, we assume that the CCEPP game is played sequentially by the patroller and the attacker. Firstly, the patroller (being the game leader) will commence a patrolling strategy $\vec{c}$ (see Formula (13), and subsequently, the attacker (being the game follower) responds with his optimal strategy accordingly (see Formula (14)). It is worth mentioning that the patrolling team is able to compute the optimal strategy of the attackers and then schedule her optimal strategy correspondingly. A Stackelberg equilibrium (SE) $\left(s_{d}^{*},s_{a}^{i*})=(\vec{c^{*}},(t^{*},k_{i}^{*},rs_{i}^{*})\right)$ for the CCEPP game is a patroller-attacker strategy pair that satisfies the following conditions:

(13) $$(t^{*},k_{i}^{*},rs_{i}^{*})=\arg\max_{(t,k_{i},rs_{i})\in S_{a}}\{u_{a}^{i}(\vec{c},(t,k_{i},rs_{i}))\},$$

(14) $$\vec{c^{*}}=\arg\max_{\vec{c^{*}}\in S_{d}}\{u_{d}(\vec{c},(t^{*},k_{i}^{*},rs_{i}^{*}))\},$$

By discretizing the time dimension, the finite number of strategies of attackers can be enumerated. For a given patroller-attacker strategy pair, payoff functions $u_{d}$ and $u_{a}^{i}$ would both be linear polynomials of $\vec{c}$. Therefore, a multiple linear programming (LP) algorithm [32] can be introduced to calculate the Stackelberg equilibrium for the CCEPP game, as shown in Table 3.

In the linear programming step, the game assists the defender to solve an LP problem. In this LP problem, the cost function is Formula (16) and the constraints are Formulas (15), (4), (5) and (6). Furthermore, the MultiLPs algorithm implements the LP step for each attacker strategy. To be specific, if the value of $c_{a}(s,e)$ is constrained to be either 0 or 1, then the optimal fixed patrolling route for the patrol team would be generated.

## 4. An Illustrative Case Study of the CCEPP Game

### 4.1. Experimental Settings

In the illustrative case, experiments are conducted in the Shanghai chemical cluster to explain how the CCEPP game works in a real industrial scene. The part areas, graphic model, and transition graph of the Shanghai chemical cluster are shown in Section 2 (i.e., Figure 1, Figure 2, and Figure 3). Further, the maximum patrolling time budget T is assumed to be 30 minutes, as the energy on the mobile patrolling vehicles is limited to a maximum of 40 minutes for driving and inspecting. Table 1 shows the patroller’s moving time between different chemical sites and inspecting time inside each site. It is assumed that patroller’s source beginning node is set to the ‘Cr2’, which means a patrol team starts her patrolling plan from this node. Some more parameters and simplification assumptions of this case are given hereafter.

For the sake of clarity, we assume that the attacker has only one release scenario to perform and this attack scenario lasts for 10 minutes with all release spots working during that period and the patrol team is assumed to follow the patrolling schedules precisely. Table 4 gives the model inputs related to the case study. They are the defender’s reward $R_{d}^{i}$ and loss ($L_{d}^{i}$) of detecting and not detecting the attacker’s irregularities; the attacker’s gain $G_{a}^{i}$ and penalty ($P_{a}^{i}$) from a successful release and from a failed release, respectively; the cost for sending a patrol team (i.e., $C_{d}=2$); and the probability $f_{cpep}$ that the release can be detected by static inspection resources. The probability that the patroller can catch the release (i.e., $\sigma_{i}$) should be provided by environmental protection authorities. However, for the sake of simplicity, we assume that if the patroller is patrolling in the $i^{th}$ chemical site and the attacker is releasing atmospheric contaminants at meantime, there is a probability of 0.05 that the attacker’s behavior would be detected by the patroller in each time slice. Moreover, the parameter ε is assumed to be 3 in this paper. The unit of all the monetary parameters can be, for instance, k¥.

It is worth mentioning that all these data concern estimations from the environmental protection authorities in the Shanghai chemical cluster. In general, the numbers of rewards ($R_{d}^{i}$), losses ($L_{d}^{i}$), and the detection probability ($f_{cpep}$) are accurate, as they are from the estimations of their own data. The amounts of rewards ($G_{a}^{i}$) and losses ($P_{a}^{i}$) for the attacker may present uncertainties because they are estimations of attackers’ data from the perspective of the defender. For instance, “The gain of a successful release in chemical site ‘A’ is 60” means that the patroller thinks the attacker will receive a value of 60 from this release. However, the study of these parameters will not be covered in this paper. For one thing, future research will consider the unknown adversaries (i.e., exact values of parameters related to the opponents are unknown). Future research can also focus on the sensitivity of the model to the values selected for parameters $R_{d}^{i}$, $L_{d}^{i}$, $G_{a}^{i}$, $P_{a}^{i}$ and ε.

### 4.2. Game Modeling 

In this illustrative case study game, there are seven players, namely a patrol team and six chemical sites. Meanwhile, the six chemical sites are independent and thus the game follows the standard paradigm of a Bayesian Stackelberg game. It is further assumed that the six chemical sites share the same prior probability (i.e., 1/6). Since only one attack scenario is considered, each attacker therefore has only m=1×30×1=30 pure strategies, being releasing excessive air pollutants at a time (i.e., at a time t∈{0,1,…,29}). The patroller has 764 possible actions, shown as edges in Figure 3. This means that the patroller’s strategy can be formed as a vector of 764 entries and each entry denotes the marginal coverage probability of the edge in the transition graph.

According to Formula (7) and (8), the patroller’s and the attacker’s payoffs can be computed. Payoffs will be represented as linear polynomials of the patroller’s strategy (i.e., $\vec{c}$), while the attacker’s strategy decides the coefficients of polynomials.

### 4.3. Results and Discussions

**Figure 4:**
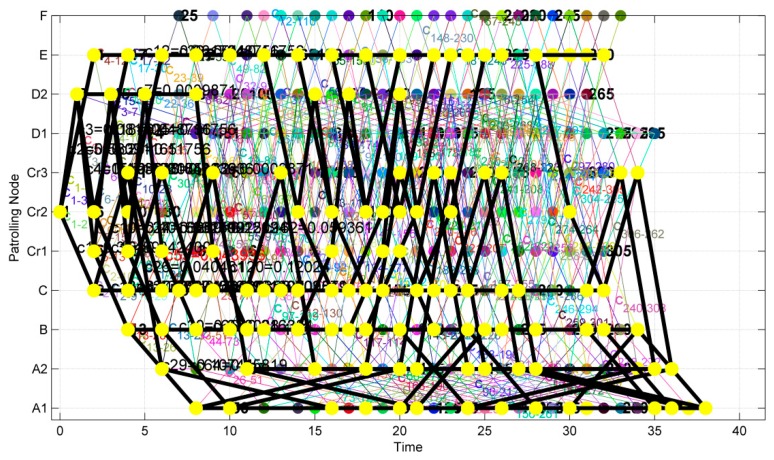
Optimal patrolling strategy generated by CCEPP game for a patrol team.

Figure 4 shows the SE of the game developed for this case, computed by the MultiLPs algorithm shown in Table 3. As we can notice, the patroller’s optimal patrolling strategy is represented by the black (and bold) lines. The associated number on the line denotes the probability that the defender will take this action. For instance, c1=0.3546 means that the patrol team should drive from the start node ‘Cr2’ to chemical site ‘C’ at a probability of 0.3546 at time 0. Similarly, c2=0.5637 denotes that at time 0, the patrol team should drive from the start node ‘Cr2’ to chemical site ‘D2’ at a probability of 0.5637. Moreover, when the patrol team is at node 1 (0, ‘Cr2’), the patroller would have three possible actions (i.e., move to chemical site ‘C’, or ‘D’, or ‘E’). The marginal coverage probabilities on the edge ‘1–2’, ‘1–3’, and ‘1–4’ are equal to the conditional probabilities of taking these actions, for the total probability in node one is 1. Furthermore, in patrolling practice, if the patroller arrives at a node in the figure, the conditional probabilities of the following actions can be calculated by the formula $c_{a}(s,out)/\sum \omega_{a}(s)$. For instance, the probability that the patroller would arrive at the node (1, ‘D2’) in Figure 4 is $\sum \omega_{a}(s)=0.5637$, and the conditional probabilities that the patroller should take the three actions (i.e., either patrolling in site ‘D’, or driving to crossroad ‘Cr2’, or crossroad ‘Cr1’) are ca1(D2,D2)=0.471550.5637=0.8365, ca2(D2,Cr2)=0.0911650.5637=0.1617, and ca3(D2,Cr1)=0.0009870.5637=0.0018, respectively. Under this optimal patrolling strategy, the payoffs for the patroller and attacker are –6.616 and 3.188 (on average), respectively. The detailed information of this optimal patrolling strategy is listed in Table A1.

Next, let us compare the SE generated by CCEPP game with the purely randomized route strategy. In real patrolling practice, patrollers often randomly schedule the patrolling route. This situation, as demonstrated in Figure 3, is simply assigning equal probabilities to edges that start from the same node. For example, when the patrol team arrives at node 1 (0, ‘Cr2’), he would move to site ‘C’, or ‘D2’, or ‘E’ with the same probability, being 1/3. However, the purely randomized patrolling strategy does not take into consideration the hazardousness level that each chemical site holds and, if this is the case, an intelligent attacker would take his preference to attack, since all the chemical sites are equally patrolled. Under this purely randomized patrolling strategy, we can firstly calculate the marginal coverage probabilities on each edge, then compute the probabilities in each overlap situation, and finally compute the payoffs of patroller and attacker. According to Formulas (7) and (8), the corresponding payoff for the patroller and attacker are –8.254 and 4.054 (on average), respectively. Compared to the SE of the CCEPP game, the defender’s payoff reduces from –6.616 to –8.254. This result reveals that the CCEPP SE strategy is characterized with a higher probability that the attacker is more possible to be discovered of his illegal behaviors. 

**Figure 5:**
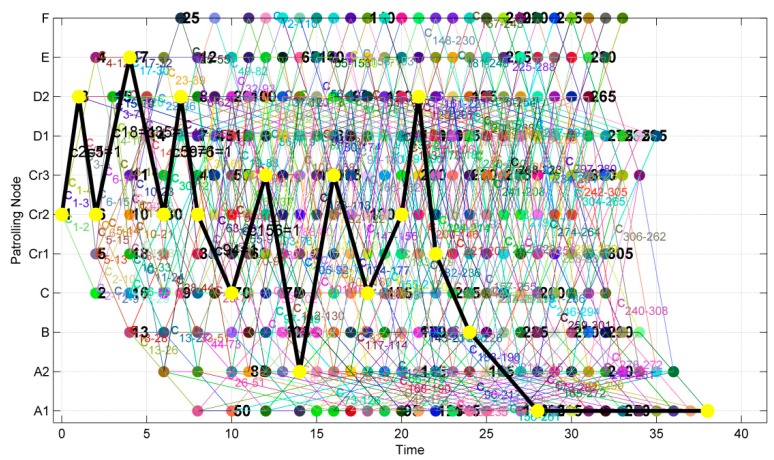
Optimal fixed patrolling route for a patrol team.

Moreover, in current patrolling practice, some patrollers may follow a fixed route strategy rather than choosing the purely randomized strategy. This scenario has been explained in detail as a black (and bold) line, as demonstrated in Figure 3. However, if a fixed patrolling route is scheduled, the patroller’s real-time location is deterministic to intelligent attackers, since intelligent attackers would collect useful information before an attack. In the transition graph, if the probability of an action being taken is further constrained to be either 0 or 1 (i.e., c∈{0,1} instead of c∈[0,1]), then a vector of that satisfies Formula (4) and (5), representing a fixed route strategy. By solving the MultiLPs algorithm, the optimal fixed route strategy is obtained, shown in Figure 5. The fixed patrolling route can be further demonstrated as: The patroller starts from ‘Cr2’; she goes to chemical site ‘D2’ and then she moves back to ‘Cr2’; she further goes to chemical site ‘E’ and then moves back to ‘Cr2’; she goes to chemical site ‘D2’ and back to ‘Cr2’ again; subsequently, she drives to chemical site ‘C’ and then moves to ‘Cr3’; she goes to chemical site ‘A2’ and moves back to ‘Cr3’; then she goes to chemical site ‘C’, crossroad ‘Cr2’, chemical site ‘D2’, crossroad ‘Cr1’, chemical site ‘B’, and chemical site ‘A1’ in sequence; finally, she patrols site ‘A’. If the patroller follows the fixed patrolling route and the attacker plays his best response, the corresponding payoff for the patroller and attacker are –8.35 and 4.15 (on average), respectively. It is worth mentioning that neither the patroller’s optimal fixed patrolling route and the attacker’s best response are unique. For instance, given the patroller’s fixed route, it would be indifferent for the attacker to start his attack at any time. However, the player’s payoff would not be different. Therefore, only one optimal fixed patrolling route is shown in this paper.

Through comparing these three strategies, the SE generated by CCEPP game obviously outperforms the purely randomized route strategy and the fixed route strategy, shown in Table 5. By implementing the SE strategy, the defender will decrease her loss and the defender’s payoff increases from −8.254 (or −8.35) to −6.616. The result reveals that the SE strategy has a higher probability of detecting attacker’s illegal releases and brings a higher reward to the defender. To be specific, higher marginal coverage probabilities would be accompanied in the more hazardous chemical sites, which implies that the holder of hazardous chemical sites is highly likely to obey air regulations and conduct permitted emissions after being caught several times. Therefore, playing a CCEPP game is essential for the patrolling team in her daily management work because the game not only improves her payoffs and detects the illegal discharge behaviors of chemical sites, but also reduces the risk of hazardous gas leakage incidents.

## 5. Conclusions

The atmospheric pollution prevention problem in a chemical cluster has drawn a great concern around the world. Though some works have been done to schedule the utilization of static inspection resources in a chemical cluster, mobile inspection resources are highly recommended to monitor the chemical production process for their flexibility and mobility. However, the current widely used patrolling strategies (i.e., purely randomized route strategy and fixed route strategy) have obvious drawbacks. To this end, a so-called chemical cluster environmental protection patrolling (CCEPP) game was developed and proposed to aid the inspection agency in effectively scheduling patrols on different chemical sites. In this game, the intelligent interactions between the patroller and the holders of chemical plants were considered. Practical road network constraints and reasonable time discretization were modeled in this game as well. Moreover, simple source estimation process in each chemical site was also considered. Finally, the MultiLPs algorithm was introduced to solve this game correspondingly. 

An illustrative case study was implemented to demonstrate how our proposed CCEPP game works in the Shanghai chemical cluster. Results of the case study show that the patroller would have higher expected payoffs by strategically randomizing patrolling routes, indicating that patrolling more potential releasing chemical plants would be more likely. These chemical sites are accompanied by higher marginal coverage probabilities. Performance of the patrolling strategy from the Stackelberg equilibrium outperforms the performances of any fixed patrolling routes and the performance of the purely randomized routes. Further, higher marginal coverage probabilities are accompanied in the more hazardous chemical sites signifying that it is more possible for the holder of hazardous chemical sites to choose to obey air regulations after several punishments. In other words, the surrounding ecosystem and residential environment will be largely improved on the one hand; the risks of hazardous gas leakage incidents will be considerably reduced by strategically scheduling the patrols on the other hand.

The proposed CCEPP game can be further modified from several directions. Firstly, the current model assumes that the estimation of the attackers’ data from the defender is correct. In reality, the model should be extended to consider unknown opponents. Secondly, the attacker is assumed to only know the probability of each action that the patroller would take. But the more realistic situation is that the intelligent adversary not only knows the probability, but also observes the current location of the patroller. To model this situation, a stochastic game is recommended. Thirdly, the patrolling inside the chemical sites is modeled simply. In reality, it is better for the patroller to determine the locations of releasing spots through source estimation methods. Therefore, future work should also focus on the application of source tracing algorithm in the CCEPP game. It would assist the patroller in tracing the releasing source and verifing this irregularity. 

## Figures and Tables

**Table 1:** Superior connection matrix for Figure 2 with the practical numbers.

Nodes	A1	A2	B	C	Cr1	Cr2	Cr3	D1	D2	E	F
A1	10	10	4	∞	∞	∞	∞	∞	∞	∞	∞
A2	10	10	∞	∞	∞	∞	2	∞	∞	∞	∞
B	4	∞	6	∞	2	∞	∞	∞	∞	∞	∞
C	∞	∞	∞	5	∞	2	2	∞	∞	∞	∞
Cr1	∞	∞	2	∞	∞	∞	∞	3	1	∞	∞
Cr2	∞	∞	∞	2	∞	∞	∞	∞	1	2	∞
Cr3	∞	2	∞	2	∞	∞	∞	∞	∞	∞	3
D1	∞	∞	∞	∞	3	∞	∞	7	7	∞	∞
D2	∞	∞	∞	∞	1	1	∞	7	7	∞	∞
E	∞	∞	∞	∞	∞	2	∞	∞	∞	6	∞
F	∞	∞	∞	∞	∞	∞	3	∞	∞	∞	7

**Table 2:** An algorithm of generating the transition graph.

Algorithm: Generating the Transition Graph
Construct an empty temporary node list *tNL*, an empty node list *tV*, and an empty edge set *tE*; Construct node *tv* = (0, *dsn*), in which *dsn* is the patrolling source node in graph *G*; Initialize *tNL* ← *tv*, *tV* ← *tv*; While *tNL* is not empty, do; Get the first node in *tNL*, denoted as the current node *cv* = (*ct*, *cn*); Construct the follow-up nodes of *cv*; In graph *G*, find all the connected nodes of *cn*, representing as *ccn* = {nd∈V|sC(cn,nd)<∞}; For each node *nd* that belongs to *ccn*, if ct+sC(cn,nd)≤T+dis(dsn,nd) holds, construct a new node *nv* = (*ct* + *sC*(*cn*, *nd*), *nd*) and a directed edge *ne* from *cv* to *nv* should also be constructed (the state transition should also be considered); Add edge *ne* to list *tE*; If *nv* is already in *tV*, continue; otherwise, insert *nv* into *tNL*, add *nv* to *tV*; Remove *cv* from *tNL*; End.

**Table 3:** MultiLPs algorithm for calculating the Stackelberg equilibrium for the Chemical Cluster Environmental Protection (CCEPP) game.

MultiLPs
***1. Initialization*** For each attacker strategy $\left(t,k_{i},rs_{i}\right)$, calculate $u_{a}^{i}$ and $u_{d}$, where are linear polynomials of $\vec{c}$; ***2. Linear Programming*** **(LP)** Suppose that the attacker strategy $\left(t^{\#},k_{i}^{\#},rs_{i}^{\#}\right)$ is the attacker’s best response, which means: $u_{a}(t^{\#},k_{i}^{\#},rs_{i}^{\#},\vec{c})\geq u_{a}(t,k_{i},rs_{i},\vec{c})\forall (t,k_{i},rs_{i})\in S_{a}$ The defender would then aim at: $Pof_{d}(t^{\#},k_{i}^{\#},rs_{i}^{\#},\vec{c^{\#}})=\max_{\vec{c}\in S_{d}}u_{d}(t^{\#},k_{i}^{\#},rs_{i}^{\#},\vec{c})$ ***3. Summary*** The Stackelberg equilibrium achieves: $\left(\vec{c^{*}},(t^{*},k_{i}^{*},rs_{i}^{*}))=\arg\max_{(t^{\#},k_{i}^{\#},rs_{i}^{\#})\in S_{a}}Pof_{d}(t^{\#},k_{i}^{\#},rs_{i}^{\#},\vec{c^{\#}}\right)$

**Table 4:** Model inputs of this case study.

	Parameter	$R_{d}^{i}$	$L_{d}^{i}$	$G_{a}^{i}$	$P_{a}^{i}$	$f_{cpep}$
Nodes						
‘A’		6	96	60	18	0.45
‘B’		6	67.2	36	18	0.3
‘C’		6	84	49.8	18	0.42
‘D’		6	72	42.6	18	0.45
‘E’		6	90	60	18	0.5
‘F’		6	78	54	18	0.4

**Table 5:** Players’ payoffs under three different patrolling strategies.

	Strategy	Stackelberg Equilibrium	Purely Randomized Route Strategy	Fixed Route Strategy
Payoff				
Defender’s payoff		−6.616	−8.254	−8.35
Attacker’s payoff		3.188	4.054	4.15

## Appendix A

**Table A1:** Defender’s strategy generated by the CCEPP game.

Edge Number	Probability	Edge Number	Probability	Edge Number	Probability	Edge Number	Probability
1	0.35459	87	0.081704	334	0.02735	525	0.072912
2	0.5637	88	0.023407	342	0.019765	526	0.20611
3	0.081704	89	0.08403	343	0.06621	532	0.095759
4	0.47155	101	0.043936	350	0.019765	554	0.042508
5	0.091165	103	0.002519	357	0.000987	560	0.068729
7	0.000987	109	0.03989	358	0.12208	561	0.077698
8	0.25294	120	0.12024	359	0.010622	569	0.01157
10	0.10165	123	0.000987	378	0.023407	571	0.008337
12	0.081704	138	0.028038	383	0.08403	574	0.095759
13	0.13548	141	0.048756	393	0.033422	586	0.025925
15	0.33607	142	0.062431	394	0.025925	587	0.043588
16	0.042409	143	0.095759	396	0.012146	592	0.00899
18	0.048756	160	0.001831	397	0.004238	606	0.13706
19	0.21851	162	0.059361	398	0.068729	616	0.035621
20	0.11756	179	0.042544	409	0.029031	619	0.01157
25	0.11756	180	0.077698	424	0.00899	626	0.068729
26	0.040461	183	0.000987	425	0.0309	633	0.040461
27	0.061192	196	0.058217	432	0.000987	646	0.077222
29	0.10744	197	0.059346	437	0.010622	648	0.015833
30	0.028038	199	0.016384	442	0.030989	650	0.01578
32	0.03989	201	0.1327	444	0.031442	656	0.00899
34	0.002519	221	0.002519	447	0.095759	664	0.068729
36	0.048756	235	0.059361	454	0.011609	676	0.20611
37	0.17457	259	0.033514	458	0.077222	688	0.066995
39	0.043936	260	0.048191	459	0.008337	690	0.00899
43	0.043936	262	0.02417	460	0.015833	699	0.032276
47	0.061192	263	0.019765	465	0.004238	701	0.072912
51	0.11756	264	0.03989	469	0.08403	731	0.030989
52	0.040461	276	0.02552	473	0.033422	744	0.072912
55	0.043936	277	0.032697	474	0.006923	750	0.035621
62	0.002519	286	0.032697	476	0.035621	751	0.01578
64	0.15819	312	0.059361	479	0.077698	756	0.015833
65	0.016384	314	0.040461	494	0.01578	757	0.27902
67	0.1327	330	0.028038	495	0.01157	762	0.01578
69	0.12024	332	0.009266	505	0.042508	763	0.063265
82	0.000987	333	0.03949	522	0.032276	764	0.27902
